# High cell-free DNA is associated with disease progression, inflammasome activation and elevated levels of inflammasome-related cytokine IL-18 in patients with myelofibrosis

**DOI:** 10.3389/fimmu.2023.1161832

**Published:** 2023-11-16

**Authors:** Geraldine De Luca, Paola R. Lev, Maria F. Camacho, Nora P. Goette, Federico Sackmann, Miguel A. Castro Ríos, Beatriz Moiraghi, Veronica Cortes Guerrieri, Georgina Bendek, Emiliano Carricondo, Alicia Enrico, Veronica Vallejo, Ana Varela, Marina Khoury, Marina Gutierrez, Irene B. Larripa, Rosana F. Marta, Ana C. Glembotsky, Paula G. Heller

**Affiliations:** ^1^ División Hematología Investigación, Instituto de Investigaciones Médicas Dr. Alfredo Lanari, Facultad de Medicina, Universidad de Buenos Aires (UBA), Buenos Aires, Argentina; ^2^ Instituto de Investigaciones Médicas (IDIM), UBA-Consejo Nacional de Investigaciones Científicas y Técnicas (CONICET), Buenos Aires, Argentina; ^3^ Laboratorio de Genética Hematológica, Instituto de Medicina Experimental, IMEX-CONICET/Academia Nacional de Medicina, Buenos Aires, Argentina; ^4^ Centro de Hematologia Pavlovsky, Fundaleu, Buenos Aires, Argentina; ^5^ Consultorios Hematológicos, Buenos Aires, Argentina; ^6^ Departamento de Hematología, Hospital Ramos Mejía, Buenos Aires, Argentina; ^7^ División Hematología Clínica, IDIM Dr. Alfredo Lanari, Facultad de Medicina, Universidad de Buenos Aires (UBA), Buenos Aires, Argentina; ^8^ Departamento de Hematología, Hospital Italiano de Buenos Aires, Buenos Aires, Argentina; ^9^ Departamento de Hematología, Hospital Universitario Austral, Buenos Aires, Argentina; ^10^ Departamento de Hematología, Hospital Italiano de La Plata, Buenos Aires, Argentina; ^11^ Departamento de Hematología, Instituto Cardiovascular de Buenos Aires, Buenos Aires, Argentina; ^12^ Departamento de Docencia e Investigación, IDIM Dr. Alfredo Lanari, Facultad de Medicina, Universidad de Buenos Aires, Buenos Aires, Argentina; ^13^ Unidad Genómica, Laboratorio Stamboulian, Buenos Aires, Argentina

**Keywords:** myelofibrosis, inflammation, NLRP3 inflammasome, cell-free DNA, IL-18, AIM2 inflammasome, JAK2, monocytes

## Abstract

Myelofibrosis (MF) is a clonal hematopoietic stem cell disorder classified among chronic myeloproliferative neoplasms, characterized by exacerbated myeloid and megakaryocytic proliferation and bone marrow fibrosis. It is induced by driver (*JAK2*/*CALR*/*MPL*) and high molecular risk mutations coupled to a sustained inflammatory state that contributes to disease pathogenesis. Patient outcome is determined by stratification into risk groups and refinement of current prognostic systems may help individualize treatment decisions. Circulating cell-free (cf)DNA comprises short fragments of double-stranded DNA, which promotes inflammation by stimulating several pathways, including inflammasome activation, which is responsible for IL-1β and IL-18 maturation and release. In this work, we assessed the contribution of cfDNA as a marker of disease progression and mediator of inflammation in MF. cfDNA was increased in MF patients and higher levels were associated with adverse clinical outcome, a high-risk molecular profile, advanced disease stages and inferior overall survival, indicating its potential value as a prognostic marker. Cell-free DNA levels correlated with tumor burden parameters and markers of systemic inflammation. To mimic the effects of cfDNA, monocytes were stimulated with poly(dA:dT), a synthetic double-stranded DNA. Following stimulation, patient monocytes released higher amounts of inflammasome-processed cytokine, IL-18 to the culture supernatant, reflecting enhanced inflammasome function. Despite overexpression of cytosolic DNA inflammasome sensor AIM2, IL-18 release from MF monocytes was shown to rely mainly on the NLRP3 inflammasome, as it was prevented by NLRP3-specific inhibitor MCC950. Circulating IL-18 levels were increased in MF plasma, reflecting *in vivo* inflammasome activation, and highlighting the previously unrecognized involvement of this cytokine in MF cytokine network. Monocyte counts were higher in patients and showed a trend towards correlation with IL-18 levels, suggesting monocytes represent a source of circulating IL-18. The close correlation shown between IL-18 and cfDNA levels, together with the finding of enhanced DNA-triggered IL-18 release from monocytes, suggest that cfDNA promotes inflammation, at least in part, through inflammasome activation. This work highlights cfDNA, the inflammasome and IL-18 as additional players in the complex inflammatory circuit that fosters MF progression, potentially providing new therapeutic targets.

## Graphical Abstract

Proposed summary scheme. High cfDNA levels participate in myelofibrosis inflammatory state, at least in part, by triggering inflammasome activation in monocytes, which results in the release of IL-18 contributing to elevated IL-18 in circulation. Levels of cfDNA and IL-18 increase progressively during disease progression and parallel systemic inflammation. Created with BioRender.com.

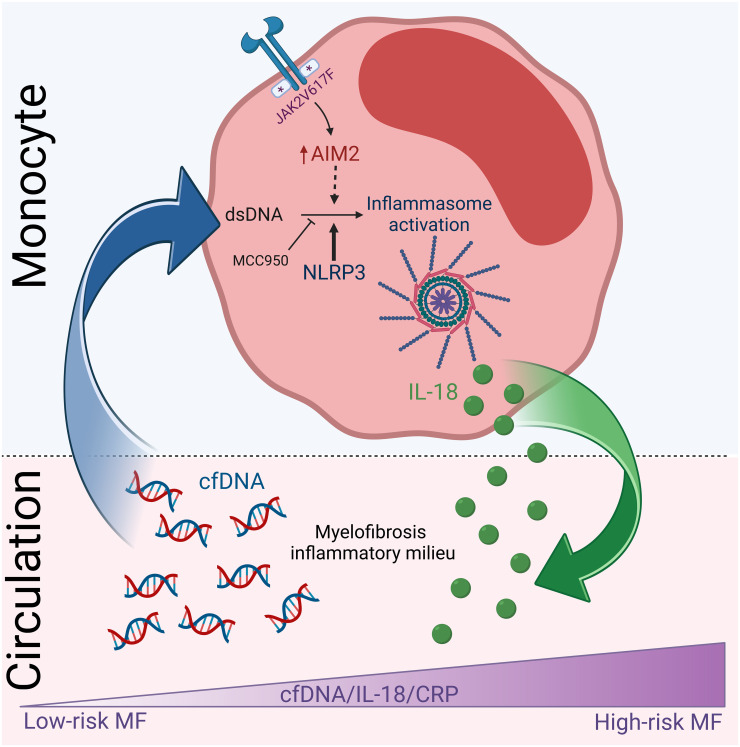

## Introduction

Myelofibrosis (MF) is a clonal hematopoietic stem cell disorder classified among chronic myeloproliferative neoplasms (MPN), characterized by exacerbated proliferation of abnormal megakaryocytes and myeloid progenitors associated with progressive bone marrow fibrosis (1). It may be primary (PMF) or secondary to polycythemia vera (PV) or essential thrombocythemia (ET), all of which share common histopathological and clinical features. Myelofibrosis is driven by *JAK2*, *MPL* or *CALR* mutations, although a small proportion of cases, so-called triple negative patients, do not harbor mutations in any of these genes. In addition, acquisition of high molecular risk (HMR) mutations influences the course of the disease, promoting clonal evolution and leukemic transformation (2). Despite substantial advances in recent years, prognosis in MF still remains poor, although it varies largely according to clinical and genetic risk factors. Individual prognosis is determined by stratification into risk groups by applying diverse prognostic models, which are based on clinical, genetic or a combination of clinical and genetic parameters (1, 3). Detrimental clinical variables include advanced age, anemia, transfusion requirement, thrombocytopenia, high leukocyte counts, increased circulating blasts and constitutional symptoms, whereas adverse genetic factors include absence of *CALR* type 1 mutation, presence of HMR mutations and unfavorable cytogenetics. Adequate patient stratification is critical for appropriate treatment decisions, which range from supporting care to allogenic stem cell transplantation (1, 3).

In close interaction with the underlying genetic abnormalities, chronic inflammation contributes to MF pathogenesis, promoting bone marrow fibrosis and clonal instability and is also responsible for clinical manifestations, such as constitutional symptoms (4–7). This inflammatory state is generated by a plethora of pro-inflammatory cytokines and chemokines released by the MPN clone but also by bystander non-clonal cells, which acquire a pro-inflammatory phenotype due to their crosstalk with mutant cells (4, 8, 9). The severity of inflammation increases along disease progression and, in this regard, certain cytokines, such as interleukin (IL)-8, IL-2R, IL-12 and IL-15, have been shown to be independently associated with an inferior outcome (8). Treatment with the JAK1/2 inhibitor ruxolitinib leads to a partial decrease in some but not all of the elevated pro-inflammatory cytokines, suggesting that other inflammatory mediators and/or signaling pathways are involved (10, 11). Damage-associated molecular patterns (DAMPs), which represent endogenous molecules released by damaged or dying cells that stimulate the innate immune response, have also been implicated in MPN inflammation, including S100A8/S100A9 (12) and fibronectin EDA isoform (13). In addition, the prototypical inflammatory factor NF-kB has been shown to be overactivated in MF as a result of cell-autonomous mechanisms and extrinsic signals, and inhibition of NF-kB signaling reduces cytokine production by MPN cells (14). The relevance of inflammation in MF pathogenesis is further revealed by preclinical data showing the therapeutic efficacy of drugs that target NF-kB, such as bromodomain and extraterminal domain (BET) inhibitors, in MPN models (15), which have led to ongoing clinical trials assessing combined BET plus JAK inhibition (16).

Cell-free (cf)DNA comprises short fragments of extracellular DNA present in small amounts in circulation mainly composed of double-stranded nuclear and mitochondrial DNA (17). Most circulating nuclear DNA is associated with histones in the form of mono- or oligo-nucleosomes. Levels of cfDNA are increased in several disease conditions, including autoimmune diseases, inflammatory conditions, prothrombotic disorders and cancer, where it has been shown to be useful as a tumor biomarker as well as to enable molecular profiling of both solid and hematologic malignancies in the context of liquid biopsy (18, 19). Double-stranded (ds)DNA behaves as a DAMP, triggering the innate immune response and promoting inflammation by stimulation of a range of specific cytoplasmic sensors, including toll-like receptor 9, the cyclic GMP-AMP synthase-stimulator (cGAS) of interferon genes (STING) pathway, that drives the production of type I interferons, the retinoic acid-inducible gene-I (RIG-I) and absent in melanoma 2 (AIM2), that leads to the assembly and activation of the AIM2 inflammasome (20–22). In addition, in primary human monocytes, dsDNA indirectly activates the NOD-, LRR- and pyrin domain-containing protein 3 (NLRP3) inflammasome by stimulating the cGAS/STING axis, which induces a lysosomal cell death program leading to K^+^ efflux, a known trigger of NLRP3 assembly (23). Inflammasomes are multiprotein platforms that mediate the activation of caspase-1, which subsequently leads to proteolytic cleavage and secretion of two key pro-inflammatory cytokines, IL-1β and IL-18, as well as activation of Gasdermin D, which leads to pyroptotic cell death (23–26). Emerging data suggests that dysregulated inflammasome function may participate in MPN inflammation (27–29).

We previously reported elevated levels of circulating nucleosomes, which represent DNA wrapped around core histones, in a limited cohort of MF patients, particularly in those with advanced disease (30). In this work, we aimed to assess the potential value of cfDNA, which represents an easy to measure parameter, as a marker of disease progression and mediator of inflammation in patients with MF.

## Patients and methods

### Patients

Seventy-two patients with MF, including overt primary myelofibrosis, post-ET and post-PV myelofibrosis, diagnosed according to the 2016 World Health Organization (WHO) criteria (31) or the International Working Group on Myelofibrosis Research and Treatment (IWG-MRT) criteria (32), respectively, were included after written informed consent. Clinical features of MF patients are summarized in **Table 1**. As controls, we studied 35 healthy individuals. Age of MF patients and controls was 66 (19–88) and 60 (26–83) years, respectively, while 56% in the MF cohort and 54% in the control group were women. In addition, patients with ET and PV diagnosed according to 2016 WHO criteria (31) were also included, as detailed in **Supplementary Table 1**. None of the patients or controls suffered from acute or chronic infections, acute thrombotic events, other neoplasms or autoimmune disease at the time of the study. This study was approved by the Instituto de Investigaciones Médicas “Dr. Alfredo Lanari” Ethics Committee.

**Table 1:** Features of patients with myelofibrosis.

	All patients (n=72)
Age (years), median (range)	66 (19-88)
Female, n (%)	40 (56%)
Myelofibrosis, n (%)	
Primary	48 (67%)
post-ET	11 (15%)
post-PV	13 (18%)
Driver mutation, n (%)	
*JAK2*V617F	39 (54%)
*CALR* type 1/type 1-like	18 (25%)
*CALR* type 2	4 (6%)
*MPL*	5 (7%)
Triple-negative	6 (8%)
High molecular risk mutation,* n (%)	
*ASXL1*	20 (28%)
*SRSF2*	5 (7%)
*IDH1/2*	1 (1%)
*U2AF1*	2 (3%)
Unfavourable karyotype** (n evaluable=43), n (%)	6 (14%)
Hemoglobin (gr/dL), median (range)	10.5 (4-17.8)
Hemoglobin <10 gr/dL, n (%)	34 (47%)
Transfusion dependency, n (%)	15 (21%)
Platelet count (x 10^9^/L), median (range)	264 (21-1626)
Platelet count <100 x 10^9^/L, n (%)	17 (24%)
Leukocyte count (x 10^9^/L), median (range)	10.6 (1.6-124)
Leukocyte count >25 x 10^9^/L, n (%)	9 (12%)
Blasts (%), median (range)	0.2 (0-7)
Blasts >1%, n (%)	29 (40%)
Constitutional symptoms, n (%)	28 (39%)
DIPSS risk score, n (%)	
low	13 (18%)
intermediate-1	26 (36%)
intermediate-2	20 (28%)
high	13 (18%)
MIPSS70 risk score, n (%)	
low	8 (11%)
intermediate	38 (53%)
high	26 (36%)
Treatment, n (%)	
None	42 (58%)
Hydroxyurea***	13 (18%)
Ruxolitinib	17 (24%)
Leukemic transformation, n (%)	5 (7%)
Death, n (%)	24 (33%)
Follow-up (months), median (range)	30.1 (1-92)

*High molecular risk mutations were detected in 25 (35%) patients, 3 of them with ≥2 variants. ASXL1 was characterized by frameshift or nonsense variants while the remaining variants comprised missense changes in IDH1 (p.R132H), IDH2 (p.R140Q), SRSF2 (p.P95H/L) and U2AF1 (p.Q157P/R).

**Unfavorable karyotype was assigned to any abnormal karyotype other than normal karyotype or sole abnormalities of 13q−, +9, 20q−, chromosome 1 translocation/duplication or sex chromosome abnormality including -Y.

***2 patients received alpha interferon in addition to hydroxyurea.

ET means essential thrombocythemia; PV, polycythemia vera; DIPSS, Dynamic International Prognostic Scoring System; MIPSS70, Mutation-Enhanced International Prognostic Score System.

Clinical and laboratory parameters were collected by chart review. Percentage of monocytes and blasts in peripheral blood were assessed by manual differential cell count. Serum lactate dehydrogenase (LDH) levels were measured by spectrophotometry. Genomic DNA samples were analyzed using allele-specific-primers for *IDH1/2* (exon 4), Sanger sequencing for *ASXL1* (exon 12-13) and high-resolution melting (HRM) confirmed by Sanger sequencing for *SRSF2* (exon 1) and *U2AF1* (exon 2) (33–37). Data on driver mutations, including *JAK2*V617F, *MPL* and *CALR*, were retrospectively available or assessed in this study by allele-specific PCR for *JAK2*V617F or Sanger sequencing for *MPL* and *CALR*.

### Plasma samples

Plasma samples were obtained from EDTA-anticoagulated blood and processed within 30 minutes of collection by two sequential centrifugation steps at 1000 x *g* at 4°C during 15 minutes each, aliquoted and stored at −80°C until assayed.

### Measurement of cell-free DNA and quantification of C-reactive protein

Cell-free DNA was quantified from plasma samples in a 10-fold dilution using Quant-iT PicoGreen dsDNA (Invitrogen, USA) in a fluorometer (BioTek Instruments, Winooski, VT, USA). Samples were also tested without PicoGreen and then subtracted to the labeled one to eliminate the plasma signal. The calibration curve was constructed using thymus DNA of a known concentration. C-Reactive protein (CRP) concentration was measured in plasma samples by human C-Reactive Protein Quantikine® enzyme-linked immunosorbent assay (ELISA) (R&D Systems, Minneapolis, MN, USA) according to the recommendations of the manufacturer.

### Isolation of monocytes

Separation of mononuclear cells from peripheral blood was performed by Ficoll-Paque gradient centrifugation (Cytiva, Sweden). Then, monocytes were isolated from peripheral blood mononuclear cells using anti-human CD14 MACS microbeads (Miltenyi Biotec, Germany), following the manufacturer´s instructions. Cell purity was routinely over 95%.

### Cell stimulation

After separation, 2 x10^5^ monocytes from each patient/control sample were seeded per well in a 48-well plate in RPMI-1640 medium with L-Glutamine, with phenol red (Life Technologies, NY, USA), supplemented with 10% human heat-inactivated serum, obtained from a healthy individual, and penicillin/streptomycin (Life Technologies). Cells were incubated for 1 h at 37°C, 5% CO2, in a humidified atmosphere to allow for attachment. For stimulation, 1 ng/mL lipopolysaccharide (LPS) derived from Escherichia coli O111:B4 (Sigma-Aldrich, St. Louis, MO, USA) alone or 1 μg/mL poly(dA:dT) (InVivoGen, California, USA) plus 1 ng/mL LPS were added to the media. Untreated cells served as control. Cationic lipid for transfecting mammalian cells LyoVec™ was added to cells in basal conditions and cells stimulated with LPS or LPS plus poly(dA:dT). In separate experiments, cells were incubated with 20ng/mL LPS alone or 20ng/mL LPS plus 10 uM Nigericin (InVivoGen, California, USA), which was added during the last 2 h. When indicated, cells were pre-incubated for 1 h with 1 uM MCC950, an NLRP3-specific inhibitor (InVivoGen, California, USA), prior to stimulation. Cells were incubated overnight at 37°C, 5% CO2, in a humidified atmosphere, and supernatants were harvested after 20 h, centrifuged at 1000 x *g* and stored at − 80°C until analysis.

### Quantification of IL-18 in plasma samples and culture supernatants

Active, cleaved IL-18 derived from monocyte culture supernatants or plasma was measured by a Quantikine®, Human total IL-18/IL-1F4 ELISA (R&D Systems) according to the recommendations of the manufacturer.

### Expression of AIM2 and NLRP3 inflammasome sensors by real-time reverse transcription polymerase chain reaction

RNA was extracted from 5 x10^5^ purified monocytes from patients and controls using Trizol reagent (Life technologies, Carlsbad, CA, USA) and reverse transcribed using SuperScript II Reverse Transcriptase (Thermo Fisher Scientific, Carlsbad, CA, USA). cDNA was analyzed by qPCR using SYBR® Green (LifeTechnologies, NY, ;USA) in a CFX96 Connect Real-Time PCR Detection System (BioRad, USA). Primer sequences were: AIM2 forward: 5’-ACTCAGCCCCTTGGAACAAT-3’; AIM2 reverse: 5’-TCCCAGTGTTGTCACTTAGGTC-3’; NLRP3 forward: 5’-TCTGTGTGTGGGACTGAAGCA-3’; NLRP3 reverse: 5’-TACTGATGCAAGATCCTGACAACA-3’. Samples were run in triplicate and assessed relative to glyceraldehyde-3-phosphate dehydrogenase (GAPDH).

### Statistical analysis

Data were tested for Gaussian distribution. For comparison between two groups, unpaired Student's *t*-test was used for normally distributed data and Mann-Whitney or Wilcoxon test were applied for non-normally distributed data. For comparison among multiple groups, Kruskal-Wallis tests with *post hoc* Dunn's Multiple Comparison test was used. Categorical values were examined by Fisher's exact test or Chi-square test. Odds ratios and 95% confidence intervals were calculated. For correlations, Spearman r coefficient was used. Overall survival was calculated from the date of sample collection to the date of death (uncensored), last contact or stem cell transplantation (censored). Kaplan-Meier curves were generated and compared using the log-rank test. Cox proportional hazard regression models were applied to calculate adjusted Hazard Ratios (HR). All statistical analyses were two-sided and *P* values < 0.05 were considered significant. The GraphPad Prism 9.3.1 (La Jolla, CA, USA) software and the STATA Software, 16.0 (College Station, TX, USA) were applied.

## Results

### Cell-free DNA is elevated in patients with myelofibrosis and is associated with adverse clinical and genetic features

**Figure 1:**
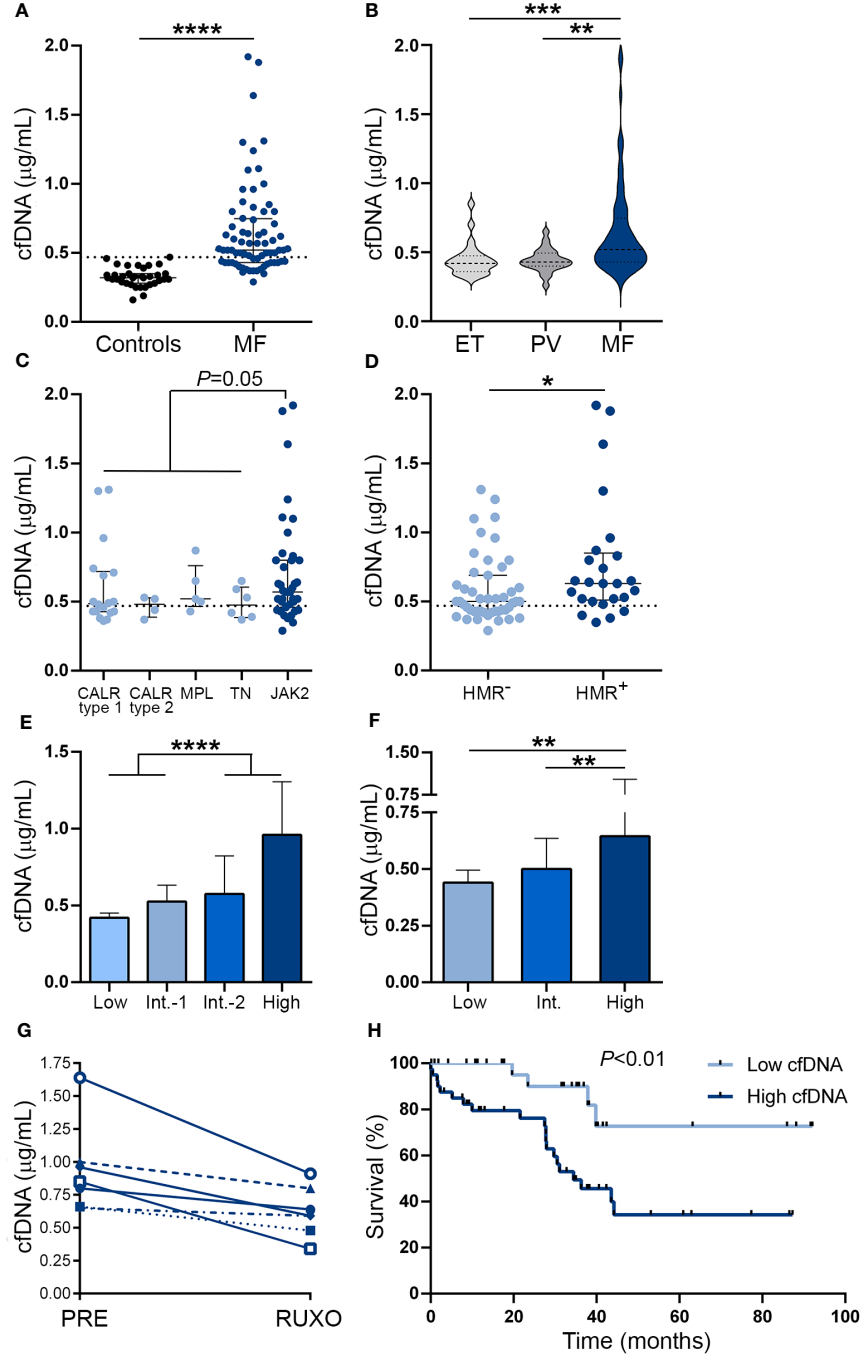
Cell-free DNA levels in patients with myelofibrosis. Cell-free (cf)DNA was quantified in plasma by fluorometry in **(A)** myelofibrosis (MF) patients (n=72) vs. controls (n=35), ****P<0.0001, Mann-Whitney test. **(B)** MF vs essential thrombocythemia (ET) (n=25) and polycythemia vera (PV) patients (n=25), **P<0.01; ***P<0.001, Kruskal-Wallis test. **(C)** MF patients grouped according to driver mutations, *CALR* type 1 (n=18), *CALR* type 2 (n=4), *MPL* (n=5) triple-negative (n=6) and *JAK2*V617F-positive (n=39) patients, *P*=0.05, Mann-Whitney test for comparison between *JAK2*V617F vs. other genotypes grouped together. **(D)** MF patients positive (n=25) or negative (n=47) for high molecular risk (HMR) mutations, *P<0.05, Mann-Whitney test. **(E)** MF patients stratified in low (n=13), intermediate (Int.)-1 (n=26), intermediate-2 (n=20) and high (n=13) risk groups according to DIPSS, ****P<0.0001, Mann-Whitney test and **(F)** low (n=8), intermediate (n=38), and high (n=26) risk groups according to MIPSS70 score, **P<0.01, Kruskal-Wallis test. **(G)** Reduction in cfDNA in sequential samples obtained prior to (PRE) and during ruxolitinib (RUXO) therapy (n=7). **(H)** Overall survival in patients stratified according to low and high cfDNA levels, *P*<0.01, log rank test. Horizontal lines **(A–D)**, bars and error bars **(E, F)** indicate the median with interquartile range. Dashed lines in **(A, C, D)** indicate reference values.

Circulating cfDNA was higher in MF patients compared to controls (**Figure 1A**). Levels were above the reference range (mean plus two standard deviations of the control population) in 67% of this MF cohort. Although cfDNA was also elevated in ET and PV compared to controls (*P*<0.01 and *P*<0.001, respectively), values were lower than in MF (**Figure 1B**). Analysis according to driver mutation status in MF revealed that *JAK2*V617F-positive patients had a trend towards higher cfDNA than patients with other driver mutations grouped together (**Figure 1C**), while patients harboring HMR mutations, including *ASXL1*, *SRSF2*, *IDH1/2* and *U2AF1*, showed significantly higher cfDNA levels in plasma (**Figure 1D**). Due to the fact that cytogenetic analysis was not available for a substantial proportion of patients included in this cohort, we used the clinical scoring system Dynamic International Prognostic Scoring System (DIPSS), and the combined clinical and genomic score Mutation-Enhanced International Prognostic Score System (MIPSS70) to classify patients into risk groups. Risk stratification according to DIPSS revealed that patients with more advanced disease (intermediate-2 and high-risk groups) displayed higher cfDNA levels compared to those in lower (low and intermediate-1) risk categories (**Figure 1E**) and similar results were obtained when patients were grouped according to MIPSS70 (**Figure 1F**).

To assess the relationship between cfDNA and clinical complications, patients were dichotomized into those with high (upper two quartiles of the patient population) and low (lower two quartiles) cfDNA values according to median values of the patient population. Features of patients grouped according to cfDNA levels are detailed in **Supplementary Table S1**. High cfDNA was associated with higher frequency of detrimental clinical variables contained in the DIPSS score, including anemia (hemoglobin less than 10gr/dL), transfusion dependency, thrombocytopenia (platelet count less than 100 x10^9^/L), leukocytosis (leukocyte counts higher than 25 x10^9^/L), increased blasts (higher than 1%) and constitutional symptoms (**Table 2**). Concerning the cytogenetic and mutational profile, patients with high cfDNA displayed higher frequency of unfavorable karyotype and HMR, whereas there was a trend for lower frequency of type 1 *CALR* mutations (**Table 2**). In accordance with the above-mentioned clinical and genetic associations, patients with high cfDNA were clustered in the DIPSS intermediate-2/high groups and the MIPSS70 high-risk category (**Table 2**).

**Table 2:** Clinical complications and adverse genetic features according to cell-free DNA levels.

	high cfDNA (%)	low cfDNA (%)	OR	95%CI	*P*
Clinical features					
Hemoglobin <10gr/dL	62.5	28.1	4.3	1.6-17.7	<0.01
Transfusion need	30.0	9.4	4.1	1.1-14.7	<0.05
Platelet count <100 x 10^9^/L	35.0	9.4	5.2	1.4-18.2	<0.05
Leukocyte count >25 x 10^9^/L	22.5	0.0	-	-	<0.01
Blasts >1%	65.0	9.4	17.9	4.9-61.1	<0.0001
Constitutional symptoms	60.0	12.5	10.5	3.0-30.9	<0.0001
Genetic features					
Unfavourable karyotype	27.3	0.0	-	-	<0.05
*CALR* type 1-negative status	85.0	62.5	3.4	1.1-11.0	0.05
HMR mutations	47.5	18.8	3.9	1.3-12.0	<0.05
Risk score					
DIPSS intermediate-2/high risk	62.5	25.0	5.0	1.8-13.4	<0.01
MIPSS70 high risk	55.0	12.5	8.5	2.4-25.2	<0.001

Patients were dichotomized into those with high (upper two quartiles) and low (lower two quartiles) cell-free (cf)DNA values. Proportions were compared using Fisher´s exact test.

cfDNA means cell-free DNA; HMR, high molecular risk; DIPSS, Dynamic International Prognostic Scoring System; MIPSS70, Mutation-Enhanced International Prognostic Score System; OR; odds ratio; 95%CI, 95% confidence interval.

Sixty percent of patients in this cohort were not receiving any treatment at the time of the study, whereas the remaining patients were treated with hydroxyurea or ruxolitinib. Cell-free DNA did not differ significantly among subgroups with different treatments, both in the overall patient cohort and when patients with lower (low and intermediate-1 groups) and higher (intermediate-2 and high) DIPSS risk categories were analyzed separately (**Supplementary Figure 1**). To avoid potential confounding effects of additional variables, we measured cfDNA levels in sequential samples from 7 ruxolitinib-treated patients, including before treatment and at 1 month after ruxolitinib was started. A significant decrease in cfDNA was evident during ruxolitinib treatment, *P*<0.05 (**Figure 1G**), along with improvement in constitutional symptoms and reduction in splenomegaly.

Median follow-up since the time of sample collection was 30.1 (1–92) months. During this period, 24 (33.3%) patients died, with an overall survival of 44.2 months. High cfDNA levels were associated with inferior overall survival on univariate analysis (hazard ratio [HR] 4.28, 95% CI 1.5-12.6, *P*<0.01) (**Figure 1H**). The effect of cfDNA on survival did not retain its significance when multivariate analysis using a Cox model that included the DIPSS score was performed (HR for high cfDNA 1.24, 95% CI 0.4-3.9, *P*=NS), and similar results were found in the context of the MIPSS70 classification (HR for high cfDNA 1.89, 95% CI 0.6-5.8, *P*=NS), reflecting the close association between cfDNA and adverse parameters included in current prognostic systems.

### Cell-free DNA correlates with tumor burden parameters and inflammatory markers

**Figure 2:**
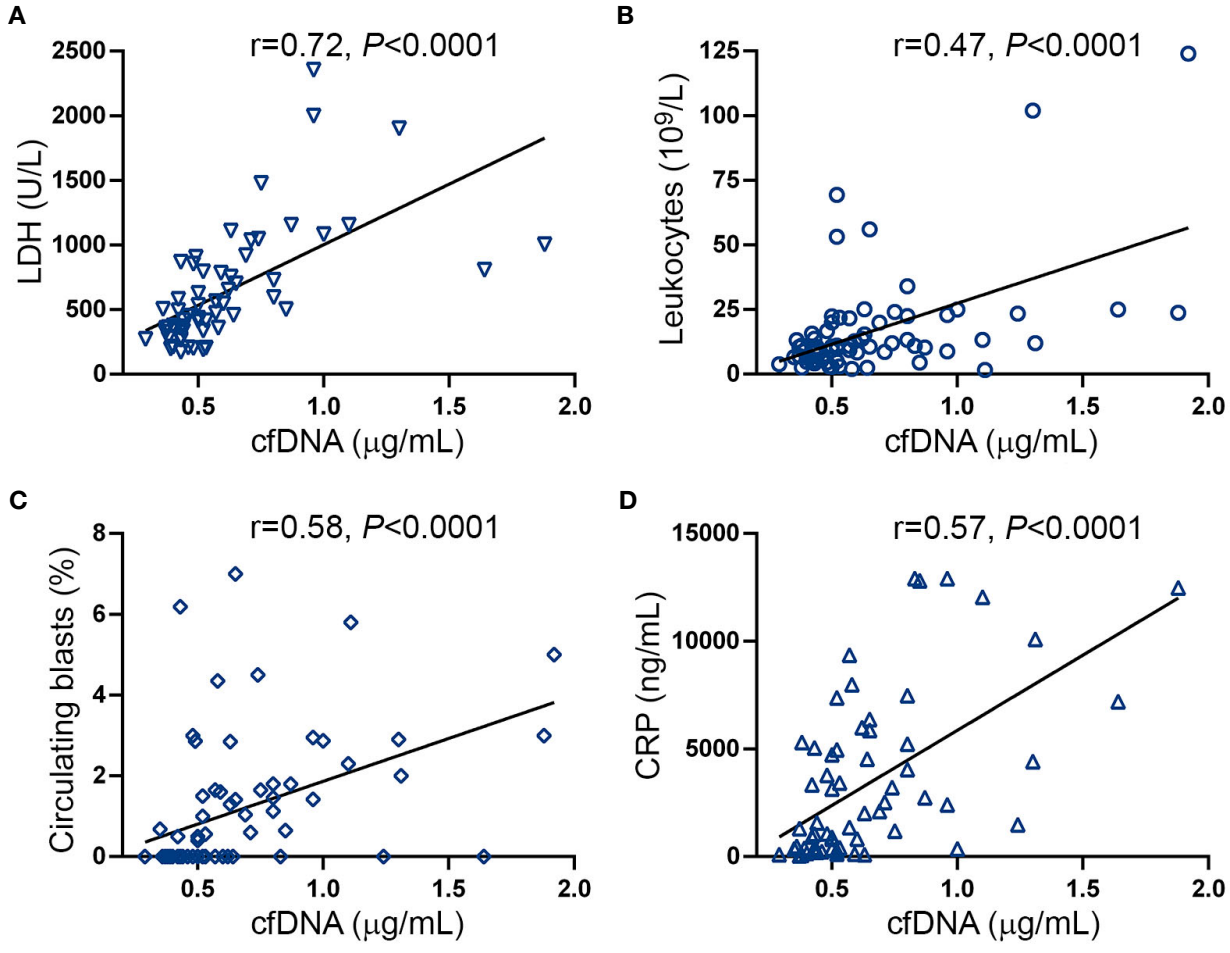
Correlation between cell-free DNA, tumor burden and inflammatory markers. Correlation between cell-free (cf)DNA and **(A)** serum LDH, **(B)** leukocyte counts, **(C)** circulating blasts and **(D)** C-reactive protein (CRP). Data were analysed using Spearman correlation.

DNA may be released to the extracellular milieu by passive or active mechanisms (17). Close relationship was found between cfDNA and serum LDH in this cohort (**Figure 2A**), suggesting that cell death, plausibly due to increased cell turnover of the MPN clone, may trigger passive DNA release into the circulation. Supporting this possibility, cfDNA correlated with tumor burden parameters, such as leukocyte counts (**Figure 2B**), circulating blasts (**Figure 2C**) and splenomegaly (spleen >5cm below the left costal margin) (OR 7.9, 95% CI 2.7-20.5, *P*<0.0001).

Disease progression in MF is fueled by sustained chronic inflammation and, accordingly, inflammatory markers increase progressively along MF evolution (38). Considering that double-stranded DNA triggers inflammation, we evaluated the relationship between cfDNA and CRP, a marker of systemic inflammation. CRP was elevated in this patient cohort (**Supplementary Figure S2A**) and, as previously reported (38), levels were higher in patients with higher DIPSS risk categories and a similar trend was found for MIPSS70 score (**Supplementary Figure S2B, C**). A close correlation was shown between cfDNA and CRP levels (**Figure 2D**), suggesting cfDNA may exacerbate the inflammatory state and, conversely, inflammation may further trigger DNA release.

### Monocytes from myelofibrosis patients show exacerbated inflammasome activation in response to double-stranded DNA

**Figure 3:**
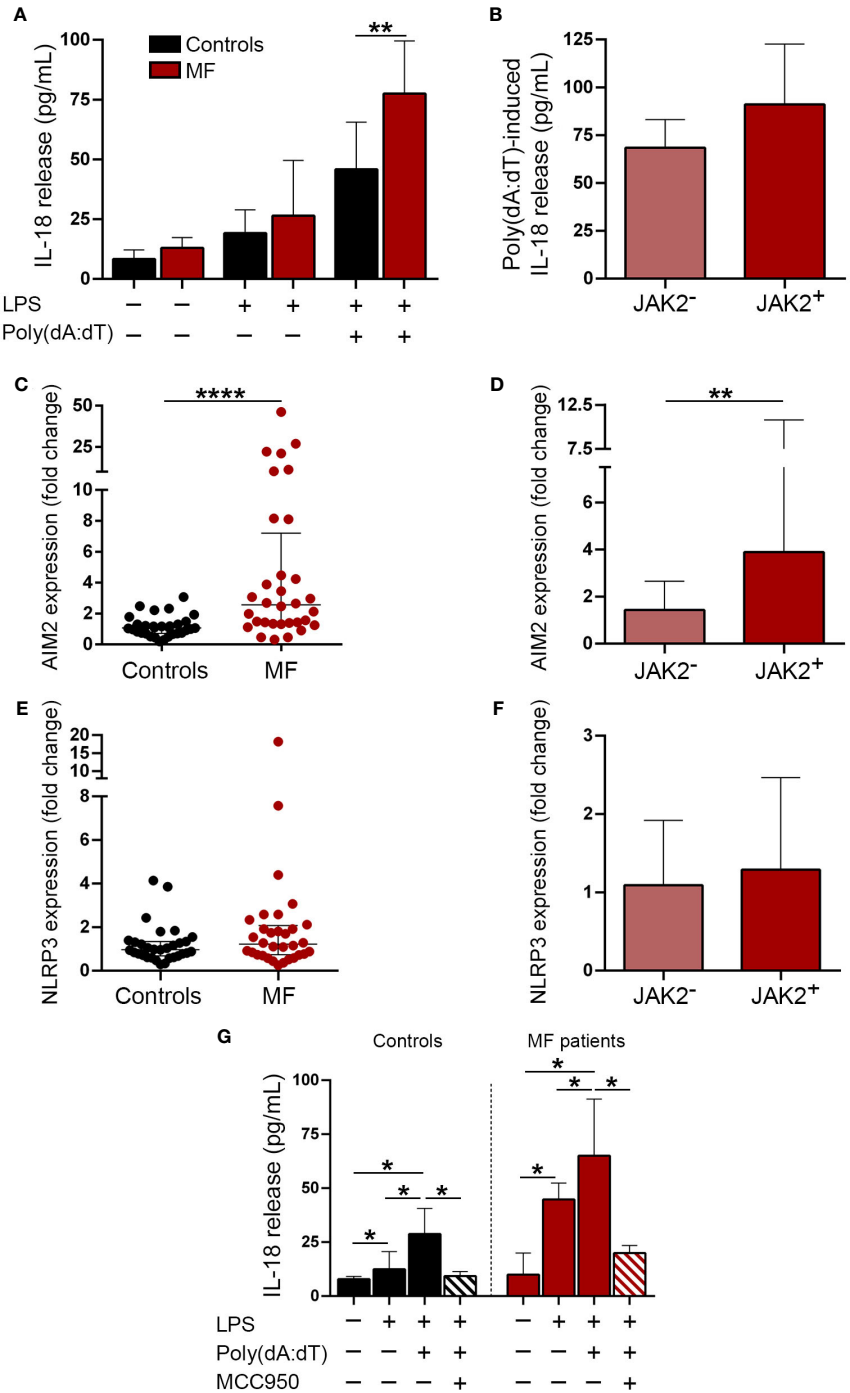
Inflammasome expression and activation in patient monocytes. **(A)** Monocytes from MF patients (n=16) and controls (n=8) were incubated under resting conditions or stimulated with LPS 1 ng/mL or LPS 1 ng/mL plus poly (dA:dT) 1μg/mL during 20 hours and IL-18 release to the culture supernatant was measured by ELISA, **P<0.01, Mann-Whitney test. **(B)** Poly(dA:dT)-induced IL-18 release in *JAK2*V617F-positive (n=9) vs. *JAK2*V617F-negative (n=7) MF patients, including those carrying *CALR* type 1 (n=4), *MPL* (n=2) mutations and triple-negative (n=1), *P*=NS, Mann-Whitney test. **(C)** AIM2 and **(E)** NLRP3 expression was assessed in purified monocytes from myelofibrosis (MF) patients (n=32) and controls (n=31) by qPCR, ****P<0.0001, Mann-Whitney test. **(D)** AIM2 and **(F)** NLRP3 levels in *JAK2*V617F-positive (n=17) vs. *JAK2*V617F-negative (n=15) MF patients, including those carrying *CALR* type 1 (n=7), *CALR* type 2 (n=1), *MPL* (n=4) mutations and triple-negative (n=3), **P<0.01, Mann-Whitney test. **(G)** Blockade of poly(dA:dT)-induced IL-18 release after incubation of monocytes with 1μM MCC950, a NLRP3-specific inhibitor. Comparison among experimental conditions in controls, *P<0.05, Wilcoxon test, and in patients (n=7), *P<0.05, Wilcoxon test. Horizontal lines **(C, E)**, bars and error bars **(A, B, D, F, G)** indicate the median with interquartile range.

Double-stranded DNA promotes inflammation by triggering several signaling pathways, including inflammasome activation (21–25). To mimic the effects of cfDNA, we incubated monocytes, which represent key mediators of inflammation in MF (11, 39), with poly(dA:dT), a synthetic dsDNA, and measured the release of inflammasome-processed cytokine, IL-18 to the supernatant after priming with low LPS concentrations. Patient monocytes released higher amounts of IL-18 after stimulation with poly(dA:dT) as compared to controls (**Figure 3A**), indicating enhanced inflammasome function. Although *JAK2*V617F-positive patients released moderately higher IL-18 levels than *JAK2*V617F-negative ones, differences did not reach statistical significance (**Figure 3B**). Although in most cell types, AIM2 is the main inflammasome assembled in response to dsDNA (25, 40), in human primary monocytes dsDNA predominantly activates the NLRP3, rather than the AIM2 inflammasome, indicating cell-specific differences (23). Levels of AIM2, which is a known JAK2-downstream target (41, 42), were elevated in MF monocytes, being higher in *JAK2*V617F-positive compared to *JAK2*V617F-negative patents (**Figures 3C, D**), whereas no significant differences were found for NLRP3 (**Figures 3E, F**). To further assess the relative contribution of AIM2 vs. NLRP3 to enhanced inflammasome function in patient monocytes, in a separate set of experiments we used the sulfonylurea derivative MCC950, a highly specific inhibitor of NLRP3 assembly (43). Incubation of MF monocytes with MCC950 blocked poly(dA:dT)-triggered IL-18 release (**Figure 3G**), indicating that, despite AIM2 overexpression, enhanced patient response to poly(dA:dT) seemed to rely mostly on NLRP3. Furthermore, stimulation of patient monocytes with higher concentrations of LPS alone or LPS plus Nigericin, classic drivers of NLRP3 assembly (44), also resulted in increased IL-18 secretion (**Supplementary Figure S3A**), further demonstrating exacerbated NLRP3 function in MF monocytes. As expected (43), prior exposure of monocytes to MCC950 blocked the response to Nigericin (**Supplementary Figure S3B**).

### Circulating IL-18, an inflammasome-processed cytokine, is elevated in myelofibrosis and correlates with cfDNA

**Figure 4:**
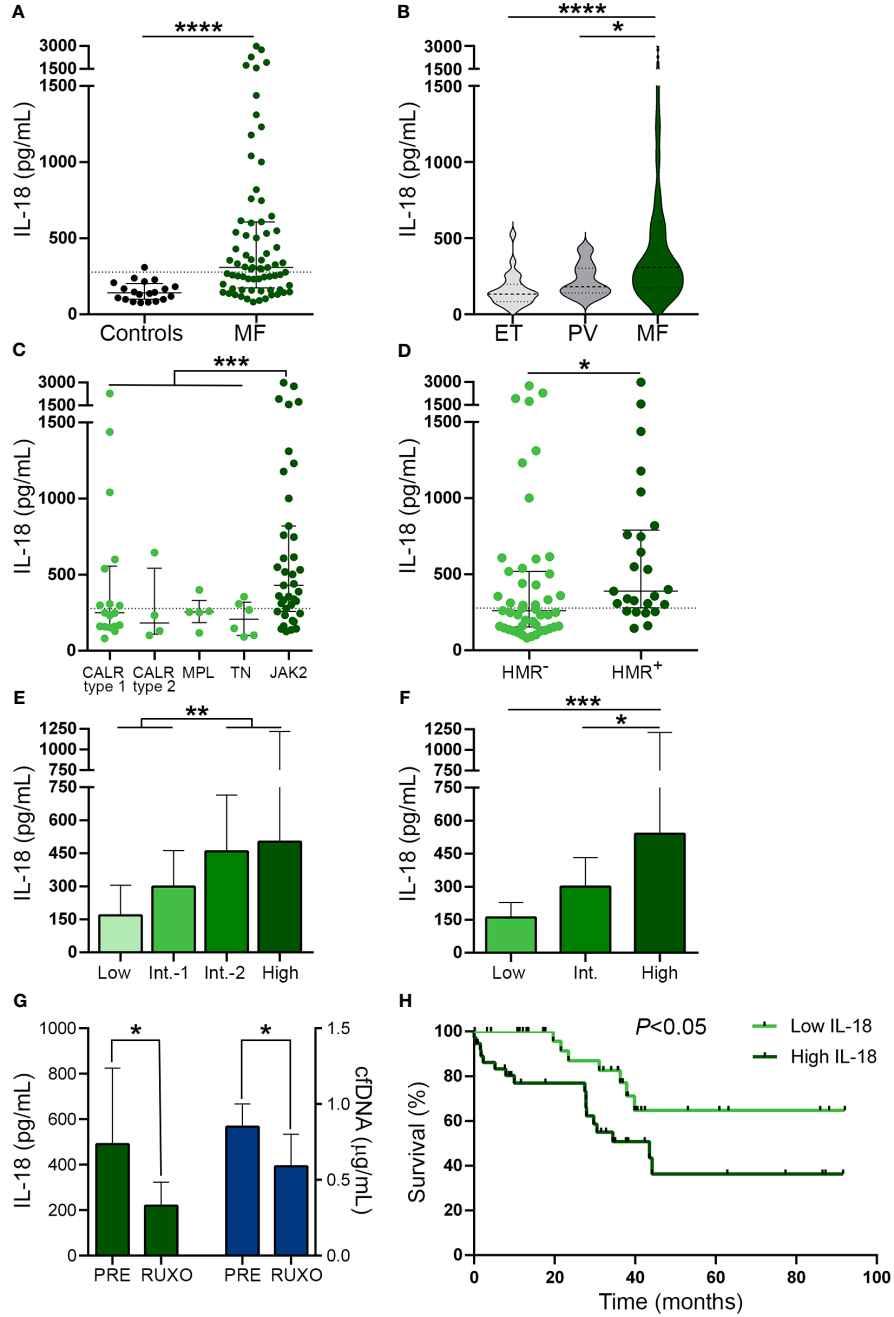
Circulating interleukin (IL)-18 levels in patients from myelofibrosis. IL-18 was measured in plasma by ELISA in **(A)** myelofibrosis (MF) patients (n=72) vs. controls (n=20), ****P<0.0001, Mann-Whitney test. **(B)** MF vs. essential thrombocythemia (ET) (n=25) and polycythemia vera (PV) (n=25) patients *P<0.05, ****P<0.0001, Kruskal-Wallis test. **(C)** MF patients grouped according to driver mutations, *CALR* type 1 (n=18), *CALR* type 2 (n=4), *MPL* (n=5) triple-negative (n=6) and *JAK2*V617F-positive (n=39) patients, ***P<0.001, Mann-Whitney test for comparison between *JAK2*V617F vs. other genotypes grouped together. **(D)** MF patients positive (n=25) or negative (n=47) for high molecular risk (HMR) mutations, *P<0.05, Mann-Whitney test. **(E)** MF patients stratified in low (n=13), intermediate-1 (n=26), intermediate-2 (n=20) and high (n=13) risk groups according to DIPSS, **P<0.01, Mann-Whitney test and **(F)** low (n=8), intermediate (n=38), and high (n=26) risk groups according to MIPSS70 score, *P<0.05, ***P<0.001 Kruskal-Wallis test. **(G)** Reduction in IL-18 in sequential samples obtained prior to (PRE) and during ruxolitinib (RUXO) therapy (n=7) in accordance with the decrease in cfDNA. *P<0.05, Mann-Whitney test. **(H)** Overall survival in patients stratified according to IL-18 levels, *P*<0.05, log-rank test. Horizontal lines **(A–D)**, bars and error bars **(E–G)** indicate the median with interquartile range. Dashed lines in **(A, C, D)** indicate reference values.

As a readout of inflammasome activation *in vivo* (45), we next measured IL-18 levels in patient plasma. Circulating IL-18 was elevated in this cohort, being above the reference range in 55% of MF patients (**Figure 4A**). To assess whether the increase in IL-18 was restricted to MF, we measured IL-18 levels in PV and ET. Although values were moderately elevated in PV (*P*<0.05), but not in ET, compared to controls, levels in MF were significantly higher than in ET and PV (**Figure 4B**). IL-18 levels were higher in MF patients harboring the *JAK2*V617F mutation vs. other driver mutations grouped together (**Figure 4C**) and also in patients with HMR mutations compared to those without (**Figure 4D**). In addition, patients with more advanced disease displayed higher levels than those belonging to lower risk categories, as assessed by both the DIPSS and the MIPSS70 risk scores (**Figures 4E, F**). No significant differences in IL-18 levels were found when patients were grouped according to treatment status (**Supplementary Figure 4**). However, as reported for other pro-inflammatory cytokines (10) and previously shown for IL-18 (46), sequential measurement of IL-18 in samples obtained prior to and at 1 month of ruxolitinib therapy (n=7) showed a reduction in IL-18 levels, mirroring the decrease in cfDNA (**Figure 4G**).

As shown for cfDNA, patients with higher IL-18 levels (upper two quartiles of the patient population) showed higher frequency of adverse hematologic parameters, clinical manifestations and HMR, and, accordingly, they were clustered into high-risk prognostic categories, compared with those with lower (lower two quartiles) levels (**Table 3**). Moreover, high IL-18 was associated with shorter overall survival, HR 2.72, 95% CI 1.1-6.6, *P*<0.05 (**Figure 4H**). The effect of IL-18 on survival did not prove to be independent of the DIPSS nor the MIPSS70 score, as estimated by multivariate analysis, adjusted HR 1.57, 95% CI 0.6-3.9, *P*=NS and HR 1.42, 95% CI 0.6-3.5, *P*=NS, respectively.

**Table 3:** Clinical complications and adverse genetic features according to IL-18 levels.

	high IL-18 (%)	low IL-18 (%)	OR	95%CI	*P*
Clinical features					
Hemoglobin <10gr/dL	62.2	31.4	3.6	1.4-9.0	<0.05
Transfusion need	29.7	11.4	3.3	0.9-10.1	0.08
Platelet count <100 x 10^9^/L	35.1	11.4	4.2	1.3-12.7	<0.05
Leukocyte count >25 x 10^9^/L	21.6	2.9	9.4	1.2-106.7	<0.05
Blasts >1%	51.3	28.6	2.6	1.1-6.6	0.06
Constitutional symptoms	56.8	20.0	5.2	1.8-13.8	<0.01
Genetic features					
Unfavourable karyotype	23.8	4.5	6.6	0.8-80.4	0.09
*CALR* type 1-negative status	83.8	65.7	2.7	0.9-8.7	NS
HMR mutations	48.6	20.0	3.8	1.3-9.9	<0.05
Risk score					
DIPSS intermediate-2/high risk	59.5	31.4	3.2	1.3-7.9	<0.05
MIPSS70 high risk	51.3	20.0	4.2	1.5-11.1	<0.01

Patients were dichotomized into those with high (upper two quartiles) and low (lower two quartiles) circulating IL-18. Proportions were compared using Fisher´s exact test.

HMR means high molecular risk; DIPSS, Dynamic International Prognostic Scoring System; MIPSS70, Mutation-Enhanced International Prognostic Score System; OR; odds ratio, 95%CI, 95% confidence interval.

**Figure 5:**
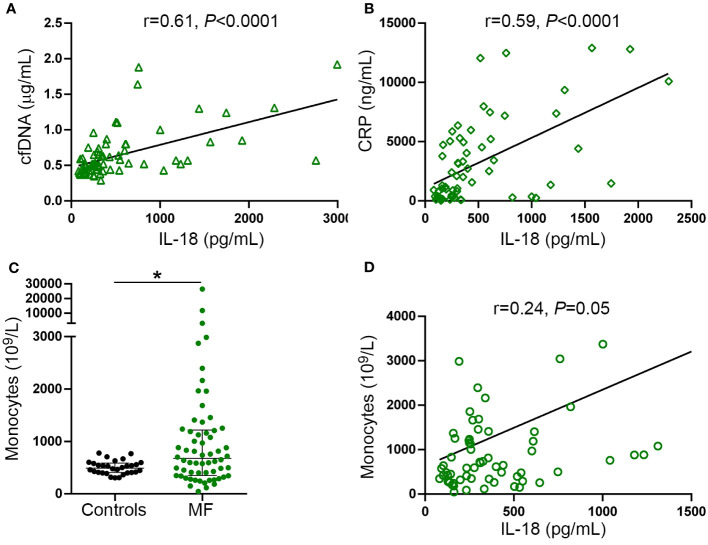
Relationship between IL-18, inflammation and cell-free DNA. Correlation between IL-18 and **(A)** cell-free (cf)DNA, **(B)** C-reactive protein (CRP). **(C)** Absolute monocyte counts in myelofibrosis (MF) patients compared to controls *P<0.05, Mann-Whitney test. Horizontal lines indicate the median with interquartile range. **(D)** Correlation between IL-18 and monocyte counts. Data were analysed using Spearman correlation.

Interestingly, tight correlation was shown between plasma IL-18 and levels of cfDNA (**Figure 5A**), both of which were proportional to the severity of the disease and paralleled systemic inflammation, as revealed by the association with CRP (**Figure 5B**). These results, together with the finding of enhanced DNA-triggered monocyte IL-18 release, suggest that cfDNA contributes to circulating IL-18 through inflammasome activation. Absolute monocyte counts were elevated in this patient cohort (**Figure 5C**) and tended to correlate with circulating IL-18 (**Figure 5D**), suggesting monocytes could represent a relevant source of IL-18 in this setting.

## Discussion

Myelofibrosis is driven by a complex interplay between driver and non-driver mutations and dysregulated inflammatory signaling that favors clonal evolution and disease progression (4–7). In this study, we measured plasma levels of cfDNA as a potential prognostic biomarker in MF and assessed its contribution to the aberrant inflammatory process that perpetuates this disease. We show that circulating cfDNA was increased in MF, being above the reference range in around two thirds of our patient population. Levels were higher in MF compared with ET and PV patients, in whom cfDNA was also increased albeit to a lesser degree. Our results are consistent with a recently published study including PV, ET and a limited number of MF patients, which showed elevated cfDNA in MPN, with highest levels in MF (47). Interestingly, the authors also show that driver and non-driver mutation status was at least equivalent in cfDNA compared to granulocyte DNA, indicating that the former sample is useful as an alternative source of DNA for molecular profiling (47).

In our MF cohort, levels of cfDNA paralleled disease evolution, increasing progressively in more advanced stages of disease. Concordantly, close association was found between higher cfDNA values and adverse clinical outcomes, including cytopenia, constitutional symptoms, splenomegaly and increased circulating blasts, with a trend for higher frequency of leukemic transformation. In addition, high cfDNA levels correlated with an adverse profile of driver (*CALR* type 1-negativity) and HMR mutations, as well as unfavorable cytogenetic abnormalities, all of which are enriched in patients with advanced MF. In agreement with the above-mentioned phenotypic and genetic correlations, patients with high cfDNA were clustered into higher risk categories according to MF prognostic scores (i.e., intermediate-2/high risk DIPSS and high-risk MIPSS70), highlighting that high cfDNA reflects a more aggressive disease course. Importantly, high cfDNA levels were associated with inferior overall survival. During the last decade, prognostic models for MF have been improved by incorporation of genomic data to clinical parameters (3). However, further refinement of contemporary scoring systems by integration of novel prognostic predictors could contribute to patient stratification for clinical decision making. As an approach to determine the value of cfDNA as an independent prognostic factor in MF, we performed a multivariate analysis in this MF cohort. Despite the effect of cfDNA on survival when considered individually, it did not retain its significance when either the DIPSS or the MIPSS70 score were included in Cox regression models. However, future studies including a larger MF cohort may be necessary to further evaluate the value of cfDNA as an independent predictor of inferior survival in MF. In this regard, marked elevation of serum LDH, a classic marker of cell death which closely correlated with cfDNA in this work, has been shown to independently predict survival in a larger MF cohort (48). Altogether, these results highlight that increased cell turnover, as assessed by high LDH or cfDNA levels, is a reflect of the degree of clonal myeloproliferation and these parameters could represent candidate biomarkers for risk stratification.

Several types of cell death may lead to the release of DNA into circulation (17). By studying patients included in this same cohort, we previously showed that *in vivo* neutrophil extracellular trap formation (NETosis), occurs in a limited subset (15%) of MF patients, as determined by detection of circulating histone-MPO complexes, which represent specific NET biomarkers (30), rendering this mechanism unlikely as a major source of cfDNA in this setting. Exacerbated proliferation and turn-over of the MPN clone leading to systemic DNA leakage is a plausible explanation, supported by the relationship between cfDNA and tumor burden parameters. Moreover, ineffective hematopoiesis, which characterizes cytopenic MF, could be regarded as a contributing factor, as suggested by the association between cfDNA and both anemia and thrombocytopenia, bearing comparison with the increase in cfDNA described in patients with myelodysplastic syndromes (49). In addition, pyroptosis, which is an inflammatory type of cell death mediated by inflammasome-induced Gasdermin D activation and membrane pore formation (26), might represent another potential mechanism. The tight relationship between cfDNA, LDH, systemic inflammation and circulating IL-18, which is released as a result of inflammasome activation, supports this possibility, which might be worth exploring in future work. Interestingly, increased inflammasome-mediated pyroptosis has recently been demonstrated in a murine *Jak2^V617F^* model (46).

In addition to the possible contribution of inflammation as a source of DNA release and considering that extracellular DNA promotes inflammation, elevated cfDNA could, in turn, reinforce the inflammatory circuit present in MF. Double-stranded DNA triggers several inflammatory pathways, including inflammasome activation and subsequent processing of inflammatory cytokines IL-1β and IL-18, as well as activation of pyroptotic executioner Gasdermin D (22–25). The role of inflammasomes, which are key players in the innate immune response, in MPN is subject of current interest (27–29). *Jak2^V617F^* macrophages from hyperlipidemic mice have been shown to display increased activation of both NLRP3 and AIM2 inflammasomes (28, 46). However, only AIM2 was overexpressed and AIM2, but not NLRP3, deletion attenuated accelerated atherosclerosis in these mice (46), pointing to a more relevant role of the AIM2 inflammasome in this model. AIM2 activation in these mice was driven by the presence of cytosolic DNA as the inflammasome trigger and, as a reflection of inflammasome function, elevated IL-18 levels were found in mouse circulation (46). In this work, we show that primary monocytes from MF patients consistently release larger amounts of IL-18 following stimulation with poly(dA:dT), a synthetic double-stranded DNA, indicating that MF patients display exacerbated inflammasome function. Consistent with the fact that AIM2 is a JAK2-downstream target (41, 42) and has been previously shown to be overexpressed in *Jak2^V617F^* murine models (46) and individuals with *JAK2*V617F-positive clonal hematopoiesis of indeterminate potential (CHIP) (50), AIM2 expression was elevated in patient monocytes, particularly in *JAK2*V617F-positive patients. Although AIM2 represents the classic cytosolic DNA inflammasome sensor and was found to be overexpressed in patients, enhanced poly(dA:dT)-induced inflammasome activation in MF monocytes seemed to rely mainly on NLRP3, as it was blocked by NLRP3 inhibition. This feature recapitulates findings in primary monocytes from healthy individuals, where, as opposed to macrophages and other cell types, dsDNA-induced inflammasome activation relies on NLRP3 (23). Whether elevated AIM2 might play a cooperating role in NLRP3 inflammasome activation in MF monocytes, as previously demonstrated for COVID19 monocytes (51), remains to be determined. In addition to poly(dA:dT), LPS plus nigericin also triggered higher IL-18 production in patients, indicating that other signals acting through NLRP3, such as S100A8/A9 (52), could contribute to NLRP3 inflammasome activation *in vivo*, as recently shown in monocytes from CMML patients (53). Considering that, besides inflammasomes, dsDNA stimulates several other inflammatory signals (20, 21), elevated cfDNA could further contribute to MF inflammation by triggering additional inflammatory cascades.

As a reflection of enhanced inflammasome function *in vivo*, IL-18 levels were elevated in patient circulation, adding IL-18 to the complex cytokine network that contributes to MF inflammatory milieu. During the preparation of this manuscript, another study carried out in a separate MF cohort also identified IL-18 among upregulated plasma cytokines, further unveiling the previously unrecognized involvement of IL-18 in MF (54). The close relationship shown here between elevated cfDNA, systemic inflammation and circulating IL-18, together with the finding of increased monocyte poly(dA:dT) response, could suggest that cfDNA triggers inflammation, at least in part, through inflammasome activation (**Graphical Abstract**). Interestingly, systemic IL-18 levels were higher in *JAK2*V617F-positive patients, consistent with prior work revealing that *JAK2*V617F is associated with increased circulating IL-18 in CHIP carriers, further linking enhanced JAK2 signaling and IL-18 (55). As described for other inflammatory cytokines (8), IL-18 levels in our MF cohort were highest in patients with more advanced stages of disease and correlated with clinical complications and decreased survival, reinforcing the concept that inflammatory mediators foster disease progression in MF.

Although multiple hematopoietic cells give rise to cytokine overproduction in MF, monocytes remain the main source of the vast majority of them (11, 39). In addition to the finding of higher monocyte IL-18 secretion, patients in this cohort had higher monocyte counts, which tended to correlate with circulating IL-18, suggesting monocytes represent a relevant source of IL-18 in this disease. However, additional cell types could contribute to IL-18 release, including neutrophils and platelets (56, 57). IL-18 has pleiotropic effects on both the innate and adaptive immune response. It is a prototypical Th1 cytokine, driving INF-γ production from T-cells, as well as other immune cells (58). It also activates macrophages, dendritic cells and monocytes, enhancing TNF and IL-1β release, and promotes neutrophil recruitment and function, acting as a general amplifier of the immune response (59). The possible functional consequences of increased IL-18 in MF patients deserve further study. In addition to IL-18 release, shown here as a read-out for inflammasome activation, other inflammasome-dependent processes, such as IL-1β secretion, have been recently shown to play an important role in MF, further strengthening the role of inflammasomes in MF pathogenesis. In this regard, two groups have recently demonstrated that genetic ablation or pharmacological inhibition of IL-1β or IL-1 receptor 1 attenuates clonal expansion and bone marrow fibrosis and osteosclerosis in *Jak2^V617F^* MPN mouse models (60, 61). In addition, as mentioned above, the potential relevance of inflammasome-related Gasdermin D activation in MF could be worth of future research.

In conclusion, we identified a novel inflammatory loop, involving circulating cfDNA, inflammasome activation and pro-inflammatory cytokine IL-18, that takes part in MF inflammation. These findings highlight that, besides previously described inflammatory cytokines, other players of the innate immune response collaborate to orchestrate the inflammatory process that contributes to MF pathogenesis, potentially providing additional therapeutic targets.

## Data availability statement

The raw data supporting the conclusions of this article will be made available by the authors, without undue reservation.

## Ethics statement

The studies involving humans were approved by Instituto de Investigaciones Medicas Dr. Alfredo Lanari Ethics Committee. The studies were conducted in accordance with the local legislation and institutional requirements. The participants provided their written informed consent to participate in this study.

## Author contributions

GDL designed and performed experiments, analyzed data, discussed results and wrote the paper; PL, NG, AG and RM discussed techniques and results, provided intellectual insight and edited the paper; MC, MG and IL performed genomic studies; FS, MCR, BM, VC, GB, EC, AE, VV, AV provided patient samples and clinical data and discussed results; MK performed statistical analyses of the data; PH conceived the study, supervised the work and wrote the paper. All authors contributed to the article and approved the submitted version.
