# Supplementary material for: High cell-free DNA is associated with disease progression, inflammasome activation and elevated levels of inflammasome-related cytokine IL-18 in patients with myelofibrosis
Source: Front Immunol. 2023 Nov 16;14:1161832. doi: 10.3389/fimmu.2023.1161832 (PMC10687201; doi:10.3389/fimmu.2023.1161832)
Supplement: Supplementary file 1 [file DataSheet_1.docx]

**Supplementary Material**

**Supplementary Tables**

**Table S1.** Features of patients with essential thrombocythemia and polycythemia vera.

ET means essential thrombocythemia; PV, polycythemia vera

**Table S2.** Clinical and molecular characteristics of MF patients stratified according to cell-free (cf)DNA levels.

Patients were dichotomized into those with high (upper two quartiles) and low (lower two quartiles) cell-free (cf)DNA values.

DIPSS means dynamic international prognostic scoring system; MIPSS70, Mutation-Enhanced International Prognostic Score System.

Data were analyzed using *t*-test or Mann-Whitney test for continuous variables and Fisher´s exact test or Chi-square for categorial variables. *P* values refer to comparison between patients with low and high cfDNA. For driver mutations, the *P* value denotes comparison between JAK2V617F-positive patients vs. all other genotypes grouped together.

**Supplementary Figures**

**
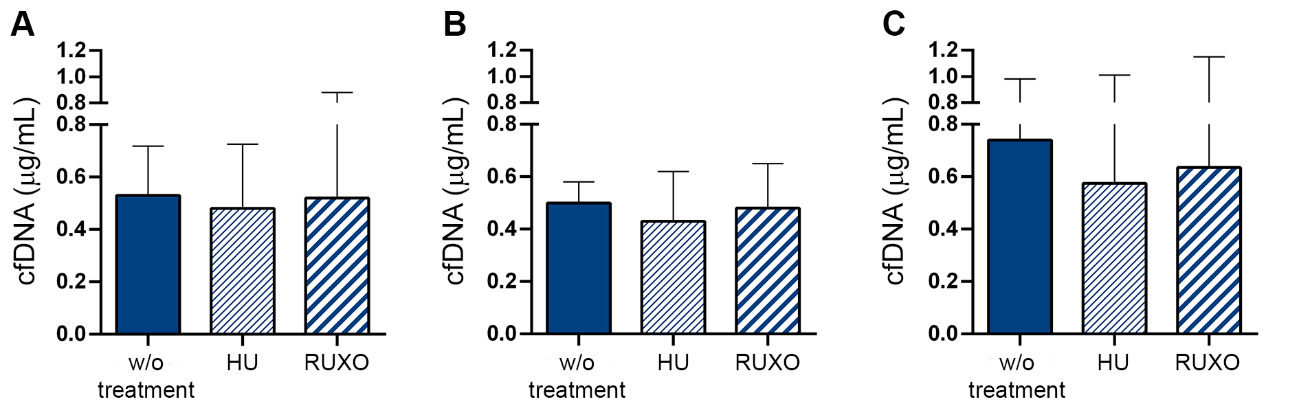
**

**Supplementary Figure S1.** **Levels of cell-free (cf)DNA in myelofibrosis patients grouped according to treatment status.** (A) Overall myelofibrosis (MF) cohort. (B) Patients with low and intermediate-1 risk according to DIPSS score. (C) Patients with intermediate-2 and high risk according to MIPSS70. Bars indicate median values with interquartile range. Differences were not significant. w/o, means without; HU, hydroxyurea, RUXO, ruxolitinib.

**
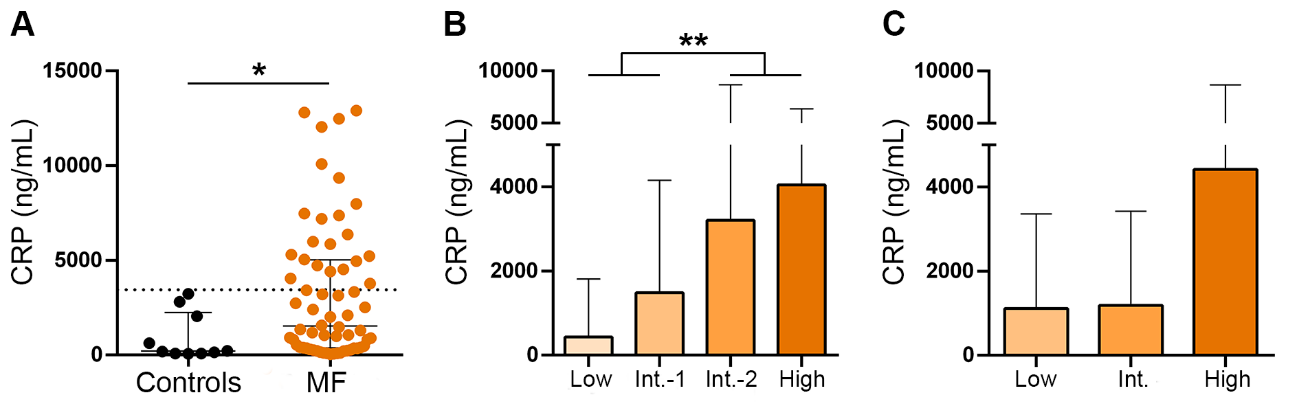
**

**Supplementary Figure S2. C-reactive protein (CRP) in myelofibrosis patients. (**A) Patients with myelofibrosis (MF) vs. controls, **P*<0.05, Mann-Whitney test. Patients stratified according to (B) DIPSS, ***P*<0.01, Mann-Whitney test and (C) MIPSS70 score, *P*=0.06, Kruskal-Wallis test. Horizontal lines (A), bars and error bars (B,C) indicate the median with interquartile range. Dashed lines indicate reference values.

**
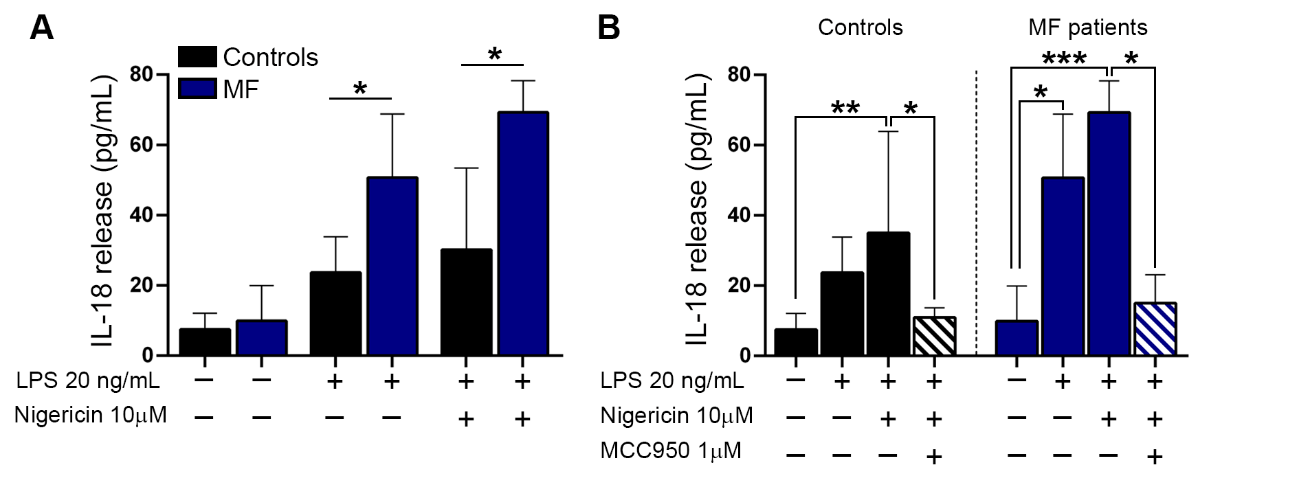
**

**Supplementary Figure S3.** **Release of IL-18 to monocyte culture supernatant after stimulation of the NLRP3 inflammasome.** (A) Monocytes from controls and MF patients (n=7) were incubated under resting conditions or stimulated with LPS 20 ng/mL or LPS 20 ng/mL plus 10μM Nigericin during 20 hours and IL-18 release to the culture supernatant was measured by ELISA, **P*<0.05 for comparison between patients and controls, Mann-Whitney test. (B) Blockade of Nigericin-induced IL-18 release after incubation of monocytes with 1μM MCC950, a NLRP3-specific inhibitor. Comparison among experimental conditions in controls, **P*<0.05, ***P*<0.01, Friedman test, and in patients (n=7), **P*<0.05, ****P*<0.001, Friedman test. Bars and error bars indicate the median with interquartile range.

**
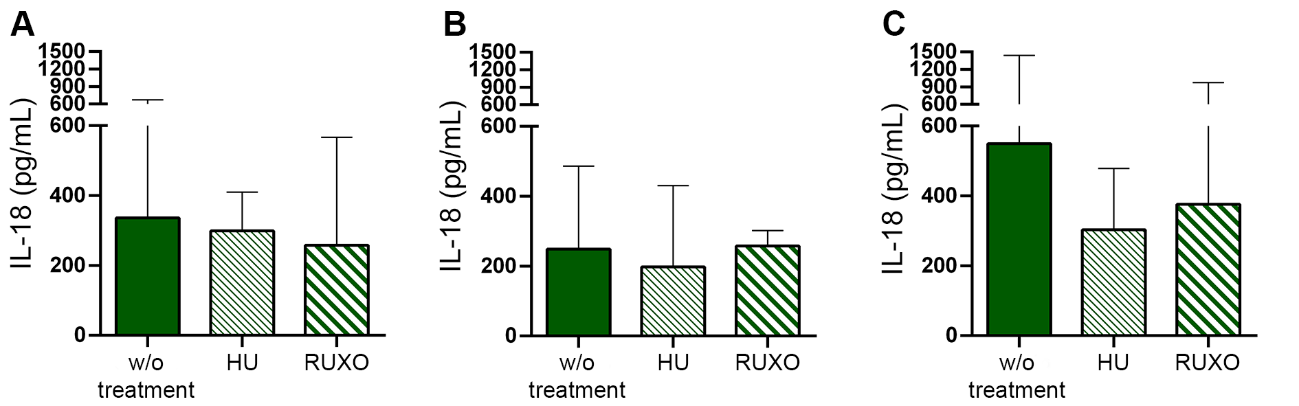
**

**Supplementary Figure S4.** **Levels of IL-18 in myelofibrosis patients grouped according to treatment status**. (A) Overall myelofibrosis (MF) cohort. (B) Patients with low and intermediate-1 risk according to DIPSS score. (C) Patients with intermediate-2 and high risk according to MIPSS70. Bars indicate median values with interquartile range. Differences were not significant. w/o, means without; HU, hydroxyurea, RUXO, ruxolitinib.
